# Conditional cash transfers and the creation of equal opportunities of health for children in low and middle-income countries: a literature review

**DOI:** 10.1186/s12939-017-0647-2

**Published:** 2017-08-31

**Authors:** Rebeca Carmo de Souza Cruz, Leides Barroso Azevedo de Moura, Joaquim José Soares Neto

**Affiliations:** 1 Postgraduate Program of Development, Society and International Cooperation of the Centre of Advanced and Multidisciplinary Studies, University of Brasilia, ICC Central, Bloco B, Mezanino, Offices 357/308, Brasilia, DF 70910-900 Brazil; 2 Faculty of Health Science and Postgraduate Program of Development, Society and International Cooperation of the Centre of Advanced and Multidisciplinary Studies, University of Brasilia, ICC Central, Bloco B, Mezanino, Offices 357/308, Brasilia, 70910-900 Brazil

**Keywords:** Child health, Equality of opportunity, Cash transfers, Social determinants of health, Equity, Policy, Development

## Abstract

**Introduction:**

Conditional Cash Transfers (CCTs) have been largely used in the world during the past decades, since they are known for enhancing children’s human development and promoting social inclusion for the most deprived groups. In other words, CCTs seek to create life chances for children to overcome poverty and exclusion, thus reducing inequality of opportunity. The main goal of the present article is to identify studies capable of showing if CCTs create equality of opportunity in health for children in low and middle-income countries.

**Methodology:**

Comprehensive literature searches were conducted in the Academic Search Complete (EBSCO), PubMed/Medline, Scopus and Web of Science electronic bibliographic databases. Relevant studies were searched using the combination of key words (either based on Medical Subject Headings (MeSH) terms or free text terms) related to conditional cash transfers, child health and equality of opportunity. An integrative research review was conducted on 17 quantitative studies.

**Results:**

The effects of CCTs on children’s health outcomes related to Social Health Determinants were mostly positive for immunization rates or vaccination coverage and for improvements in child morbidity. Nevertheless, the effects of CCTs were mixed for the child mortality indicators and biochemical or biometric health outcomes.

**Conclusions:**

The present literature review identified five CCTs that provided evidence regarding the creation of health opportunities for children under 5 years old. Nevertheless, cash transfers alone or the use of conditions may not be able to mitigate poverty and health inequalities in the presence of poor health services.

**Electronic supplementary material:**

The online version of this article (doi:10.1186/s12939-017-0647-2) contains supplementary material, which is available to authorized users.

## Background

Conditional cash transfers (CCTs) are regular money transfers to poor households given under conditions related to the use of health services, the uptake of food and nutritional supplementation, the enrollment and attendance of children and adolescents in school [1]. CCTs were initially implemented in Mexico, Brazil and Bangladesh in the 1990s, but they have been largely used in the world during the past decades, including programs initiated in more advanced economies such as the US [2].

Their success is based on the assumption that they are able to enhance children’s human development [3] through improvements of health and schooling of poor and vulnerable children, contributing to breaking the intergenerational poverty cycle [4] and social inclusion of the deprived groups [5]. In other words, CCTs seek to create life chances for children to overcome poverty and exclusion, thus reducing inequality of opportunity.

### Inequality of opportunity in health and CCTs

Inequality of opportunity is concerned with the outcome disparities sourced by factors considered unfair which are defined as circumstances exogenous to the individuals [6, 7], such as parental socioeconomic characteristics, financial hardships in early childhood, and birth characteristics which include sex, race and ethnicity [8, 9]. From this perspective, individuals with the same circumstances are aggregated into social groups that indicate a situation in which some are more privileged than others. Therefore, equality of opportunity is achieved when opportunities between social groups are equally distributed [7], so their differences in outcome are not influenced by circumstances, but from individual aspects alone [8].

For the specific case of health, inequality of opportunity is related to what is known as health inequities. This concept defines that avoidable inequalities in health are fueled by the Social Determinants of Health (SDH), which are “the conditions in which individuals are born, grow, work, live, and age” [10]. Health inequalities sourced by SDH are seen as the root for the great discrepancies in health status in the world [11], demanding the increase of equitable initiatives [12] such as CCTs.

### Purpose and context of the present study

The main goal of the present study is to identify studies capable of showing if CCTs create equality of opportunity in health for children under 5 years in low and middle-income countries. Our research question is “Do CCTs promote equality of opportunity in health for children under five years old in low and middle- income countries?”.

We focus on the specific case of child health because it remains a major public health concern in low and middle-income regions [3]. In addition, children in the first 5 years of life living in these countries usually face multiple health risks [13], which can hamper child development and negatively influence an individual’s life course [14]. For this reason, CCTs seems to be an effective social intervention capable of addressing immediate and underlying causes of poor child health, since they have presented positive effects in the use of healthcare services and income deprivation by beneficiary children [15]. Hence, the provision of better access to health by CCTs should create equality of opportunity in health if they reduce the influence of health inequities on the health outcomes of vulnerable groups, especially the younger generations.

Finally, studies concerning inequality of opportunity in health have been usually explored in more developed settings [16], but since CCTs have emerged as a key equitable policy in low and middle- income countries [2], it is time to understand if these programs create equality of opportunity in health for children in these locations.

## Methods

An integrative literature review was conducted with the purpose to comprehend if CCTs create equality of opportunity in health for children. Differently from other types of reviews, this method discusses and summarizes a particular topic, contributing to theory development and influencing practice and policymaking [17] An integrative research review was conducted because a meta-analysis was not feasible due to the heterogeneity of the studies.

### Eligibility criteria

For the purpose of the present study, the promotion of equality of opportunity in health is identified when there are improvements in health outcomes of children under 5 years old related to SDH and enrolled in a CCT program. We identified the SDH during the phase of the full-article reading.

We defined eligible health outcomes to be: (i) biochemical or biometric health outcomes with recognized relationships to illnesses or health conditions, such as height, weight, BMI, hemoglobin A1C, etc. (ii) measures of disease incidence, prevalence, morbidity and mortality; (iii) reported general health status, and (iv) utilization of health services.

We focused specifically on children from 0 to 5 years old since this life stage is critical for child development and life course [13]. Therefore, studies focusing on the effects of CCTs on the health of children from 5 to 9 years old, adolescent health, adult health and elderly people’s health were excluded. Low and middle-income countries were classified according to the cut-off criteria proposed by the World Bank [18].

Studies have been published between January of 2006 and June of 2016 in English, Portuguese and Spanish, with quantitative or mixed methods. Qualitative work, case reports, conference papers, book chapters, books, study protocols, papers not including original data such as editorials, letters to the editor and commentaries were excluded. The same applies to the studies that were not published as full reports. Thirteen reviews were retrieved from our search, but they were excluded since they did not present any results specifically for our targeted ages, did not have any of our desired child health outcomes or the results were included in a larger revision of financial incentive programs in health, so we were unable to separate their effects from other interventions.

We have included only peer-reviewed work, so no gray literature was considered. Nevertheless, in the case where we could not access the original study from databases, we considered a previous version of the article available online as a working or discussion paper. In the end, only two studies could not be accessed.

### Search methods for identification of studies

Comprehensive literature searches were conducted in the electronic bibliographic databases Academic Search Complete (EBSCO), PubMed/Medline, Scopus and Web of Science. Relevant studies were searched using the combination of key words (either based on MeSH terms or free text terms) related to conditional cash transfer, child health and equality of opportunity.

In addition, studies were also identified through consulting selected articles’ references lists. More detailed description of the search terms is available in Additional file 1.

### Data collection and analysis

**Fig. 1:**
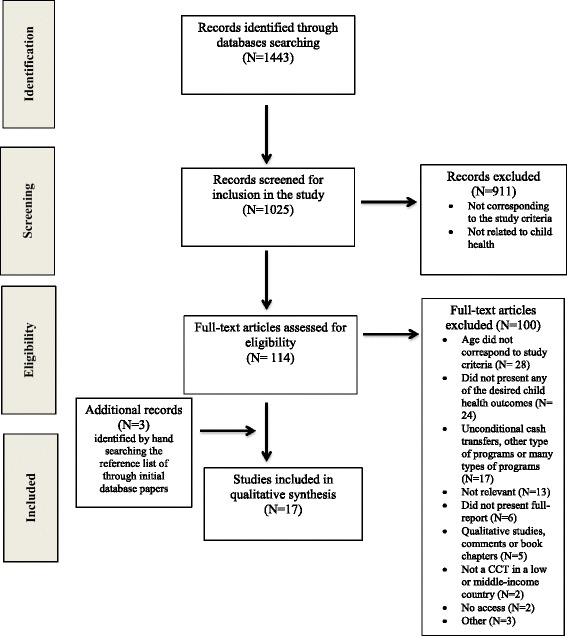
Literature selection process

The literature selection process was based on screening the title and abstract of the searched results. After applying the inclusion and exclusion criteria, a full-article reading was conducted in order to identify eligible articles. During this stage, additional literature was identified through consulting selected articles’ references lists. The flow of studies through the selection process is detailed in Fig. 1 according to Prisma guidelines [19].

Two researchers [RCBS and LBAM] screened all titles and abstracts and also participated in the selection process. Discrepancies were resolved by discussion by these two researchers, until agreement on inclusion or exclusion of the study was reached. The final sample contained articles that had classified data according to methodological research design, CCT program, country and related children’s health outcomes in Table 1.

**Table 1:** Selected Studies, CCT program, country, study design, SDH associated to beneficiaries and health outcomes

No.	Title of study	CCT Program	Country	Design	SDH associated with beneficiaries	Health outcomes	Main results
1	The impact of Mexico’s conditional cash transfer programme, Oportunidades, on birthweight [37]	Oportunidades	Mexico	Experimental	Low-income and underpriviliged locations (rural area), indigenous background	Estimation of the impact of the programme on birthweight in grams and low birth- weight (<2500 g)	Oportunidades beneficiary status was associated with higher birthweight (127.3 g) among participating women and a 4.6 percentage point reduction in low birthweight.
2	Empowering women: how Mexico’s conditional cash transfer programme raised prenatal care quality and birth weight [38]	Oportunidades	Mexico	Experimental	Low-income and underpriviliged locations (rural area), indigenous background	Estimation of the impact of the programme on birthweight in grams and low birth- weight (<2500 g)	Study showed that birth weight of beneficiaries are on average 127.3 g higher than non-beneficiaries and that the incidence of low birth weight is 44.5% lower among beneficiaries. They also found that the improvement in birth outcomes is entirely explained by better quality of prenatal care.
3	A healthier start: The effect of conditional cash transfers on neonatal and infant mortality in rural Mexico [24]	Progresa (later called Oportunidades)	Mexico	Experimental	Recipients had higher illiteracy rates and less access to electricity.	Evaluation of the impact of the Mexican conditional cash transfer program on infant and neonatal mortality	Progresa led to a large 17% decline in rural infant mortality among the treated, but did not reduce neonatal mortality on average. The benefit–cost ratio is between 1.3 and 3.6. Tests for heterogeneity show larger declines for some groups including those municipalities whose pre-program levels of mortality were above the median, and those that prior to the program had higher illiteracy rates, and less access to electricity.
4	Eradicating diseases: The effect of conditional cash transfers on vaccination coverage in rural Nicaragua [21]	Red de Proteccion Social	Nicaragua	Experimental	Low-income and underpriviliged locations, mother with a low educational level	Evaluation of the impact of the Nicaraguan program on vaccination coverage	Double-difference estimates showed the program led to large increases in vaccination coverage, and these resulted in vaccination levels greater than 95% for some vaccines
5	More evidence on the impact of India’s conditional cash transfer program, Janani Suraksha Yojana: Quasi-experimental evaluation of the effects on childhood immunization and other reproductive and child health outcomes [20]	Janani Suraksha Yojana	India	Quasi-experimental design	Low-income and underpriviliged locations, ethnicity background	Evaluation of the impact of JSY on immunization rates	Receipt of financial assistance from Janani Suraksha Yojana led to an increase in immunization rates ranging from 3.1) percentage points for one dose of polio vaccine to 9.1 percentage points in the proportion of fully vaccinated children.
6	Nutritional condition of children who benefit from the “Bolsa Família” programme in a city of northwestern São Paulo State, Brazil [31]	Bolsa Familia	Brazil	Observational, cross-section study	Low-income location	Assessment of Height for age, Weight for age, Weigh for height, BMI for age in z-scores for children enrolled in Bolsa Familia	8.8% of the children havedeficits concerning height/age and 4.2% have deficits concerning weight/age; 8.1% and 7.4% are overweight concerning weight/age and weight/height; 4.6% of the children under 2 years old have higher weight than the expected for their age and also for their height, and 7.8% of the children have low height for their age. The prevalence of weight deficit and excess in children observed in thisstudy were similar to those found in other regions of Brazil.
7	Estado nutricio de dos generaciones de hermanos(as) < de 5 años de edad beneficiarios(as) de Oportunidades, en comunidades rurales marginadas de Chiapas, México [39]	Oportunidades	Mexico	Observational, cohort study	Low-income and underprivileged location (rural area), ethnicity	Assessmeent of Height for age, Weight for age, Weigh for height of children enrolled in Oportunidades	43.4% of brothers and sisters evaluated in 2010–2011 showed stunting, underweight prevalence declined from 18% to 13.2%, wasting (low weight for height) increased from 8.1% to 10.4%. Overweight and obesity increased significantly by 12 percentage points among brothers and sisters, from 24.8% in 2002–2003 to 36.8% in 2010–2011.
8	The Combined Effects of the Expansion of Primary Health Care and Conditional Cash Transfers on Infant Mortality in Brazil, 1998–2010 [25]	Bolsa Familia	Brazil	Mixed ecological design, combining an ecological multiple-group design with a time-trend design	Low-income location	Evaluation of the effects of Bolsa Familia on postneonatal mortality	The association of higher Family Health Program (FHP) coverage with lower postneonatal infant mortality became stronger as BFP coverage increased.
9	Child health in rural Mexico: Has progresa reduced children’s morbidity risks? [23]	Progresa (lately called Oportunidades)	Mexico	Experimental	Low-income location	Impact of Progresa on child Morbidity (incidence of diarrhoea and acute respiratory infections)	Progresa contributed to reducing morbidity rates. The authors found that for both diseases under study, the Programme effect was mainly due to a decrease in the morbidity risks of children aged between 24 and 59 months. However, the evidence of a Programme effect was stronger for diarrhoea than for respiratory infections.
10	Role of cash in conditional cash transfer programmes for child health, growth, and development: an analysis of Mexico’s Oportunidades [40]	Oportunidades	Mexico	Experimental	Households with a low-income level	Impact of Oportunidades on Height for age, Weight for age, Weigh for height, BMI for age, haemoglobin concentration, number of sick days in the 4 weeks before the survey	A doubling of cash transfers was associated with higher height-for-age Z score (β 0.20, 95% CI 0.09–0.30; *p* < 0·0001), lower prevalence of stunting (−0.10, −0.16 to −0.05; *p* < 0·0001), lower body-mass index for age percentile (−2.85, −5.54 to −0.15; *p* = 0.04), and lower prevalence of being overweight (−0.08, −0.13 to −0.03; *p* = 0.001). A doubling of cash transfers was also associated with children doing better on a scale of motor development, three scales of cognitive development, and with receptive language.
11	India’s Janani Suraksha Yojana, a conditional cash transfer programme to increase births in health facilities: an impact evaluation [28]	Janani Suraksha Yojana	India	Observational, case-control study	Low-income women with low- educational levels, ethnicity	Effects of JSY on child mortality (perinatal deaths per 1000 pregnancies and neonatal deaths per 1000 livebirths)	The poorest and least educated women did not always have the highest odds of receiving JSY payments. JSY had a significant effect on increasing antenatal care and in-facility births. In the matching analysis, JSY payment was associated with a reduction of 3.7 perinatal deaths per 1000 pregnancies and 2.3 neonatal deaths per 1000 livebirths. In the with-versus-without comparison, the reductions were 4.1 perinatal deaths per 1000 pregnancies and 2.4 neonatal deaths per 1000 livebirths
12	Effects of a conditional cash transfer programme on child nutrition in Brazil [29]	Bolsa Familia	Brazil	Observational, cross-section study	Low-income and underpriviliged locations, low-educational level of the head of the family, female headed household, houses with lack of piped water and electricity	Effect of Bolsa Familia on height for age, weight for age and weight for height z-scores	Children from families exposed to the BFP were 26% more likely to have normal height for age than those from non-exposed families; this difference also applied to weight for age. No statistically significant deficit in weight for height was found. Stratification by age group revealed 19% and 41% higher odds of having normal height for age at 12–35 and 36–59 months of age, respectively, in children receiving Bolsa Familia, and no difference at 0–11 months of age.
13	Effect of a conditional cash transfer programme on childhood mortality: a nationwide analysis of Brazilian municipalities [26]	Bolsa Familia	Brazil	Mixed ecological design, combining an ecological multiple-group design with a time-trend design	Municipalities with a lower socioeconomic status	Effects of Bolsa Familia on under-5 mortality rate	Under-5 mortality rate, overall and resulting from poverty-related causes, decreased as BFP coverage increased. The rate ratios (RR) for the effect of the BFP on overall under-5 mortality rate were 0.94 for intermediate coverage, 0.88 for high coverage, and 0.83 for consolidated coverage. The effect of consolidated BFP coverage was highest on under-5 mortality resulting from malnutrition (RR 0.35) and diarrhoea (0.47).
14	Effects of unconditional and conditional cash transfers on child health and development in Zimbabwe: a cluster-randomised trial [22]	Manicaland HIV/STD Prevention Project	Zimbabwe	Experimental	Low-income level, having orphans or being child-headed or eldery-headed household or having ill or disabled household member	Impact of the CCT on the proportion of chilren younger than 5 years with up-to-date vaccinations	The proportions of children aged 0–4 years with complete vaccination records was 3.1% greater in the UCT group and 1.8% greater in the CCT group than in the control group.
15	Brazil’s Conditional Cash Transfer Program Associated With Declines In Infant Mortality Rates [27]	Bolsa Familia	Brazil	Mixed ecological design, combining an ecological multiple-group design with a time-trend design	Municipalities with lower levels of child development and health services coverage	Association of Bolsa Familia on child mortallity rates (infant mortality rate, neonatal and postnatal mortality rate)	During the first five years of the program, BFP was associated with a significant 9.3% reduction in overall infant mortality rates, with greater declines in postneonatal mortality rates than in mortality rates at an earlier age and in municipalities with many users of Brazil’s Family Health Program than in those with lower use rates. There were also larger effects in municipalities with higher infant mortality rates at baseline.
16	Financial incentives in health: New evidence from India’s Janani Suraksha Yojana [40]	Janani Suraksha Yojana	India	Observational, cohort study	Low-income women with low- educational levels, ethnicity	Effects of JSY on neonatal or early neonatal mortality	The results showed that cash incentives to women were associated with increased uptake of maternity services but there is no strong evidence that the JSY was associated with a reduction in neonatal or early neonatal mortality.
17	Anthropometric assessment and food intake of children younger than 5 years of age from a city in the semi-arid area of the Northeastern region of Brazil partially covered by the bolsa família program [30]	Bolsa Familia	Brazil	Observational, cross-section study	Low-income locations and households with a lower probability of water supply	Assessing weight-for-age, height-for-age and weight-for-height z-scores of children enrolled in Bolsa Familia	Of the studied children, 4.3% were underweight, 9.9% were stunted and 14.0% were overweight. The nutritional status of children whose families receive the Bolsa Família financial aid was not significantly different from those whose families do not receive the aid. In both groups, the consumption of fruits and non-starchy vegetables was low and similar. Children from families who receive the aid were three times more likely to eat junk food.

## Results

The literature search resulted in 1443 papers, including empirical and theoretical studies. During the second phase of the literature selection process, 114 articles were fully screened, and 17 articles were selected for analysis after applying the inclusion and exclusion criteria (Fig. 1). The majority of selected articles focused on CCTs programs located in Latin America (*n* = 13) following studies about CCTs based in Asia (*n* = 3) and Africa (*n* = 1) (Table 1). Moreover, the majority of the articles were delineated as quasi-experimental and experimental study designs (*n* = 8) and six papers were classified as observational studies. There were also studies that used a mixed ecological design, combining an ecological multiple-group design with a time-trend design (*n* = 3) (Table 1).

The articles presented studies of five CCT initiatives. Eighty two percent of the articles presented CCTs programs (*n* = 14) focused in alleviating household immediate poverty and in improving children’s health or/and schooling status by transferring cash to participants under the condition of children’s school attendance, health visits and, in some cases, the attendance of health education talks (Table 2). The remaining articles (*n* = 3) were based on the Janani Suraksha Yojana program, a CCT designed to prevent maternal and neonatal mortality by providing cash to vulnerable women at the time of delivery (Table 2).

**Table 2:** Health conditionalities of the CCT programs

No.	CCT Program	Country	Health Conditionalities
1	Oportunidades (previously called Progresa and currently named as Prospera)	Mexico	The cash transfers are conditional on every household member’s participation in three important health activities: growth monitoring from conception to age 5; regular preventative health check-ups for all family members, including prenatal care and immunizations, and; mother’s attendance at health, hygiene and nutrition education talks.
2	Bolsa Familia	Brazil	The cash transfers are given under the conditions of complying with a health and nutrition agenda, including antenatal care, vaccination, health and nutrition monitoring
3	Janani Suraksha Yojana	India	Eligible women receive cash assistance upon delivering in an accredited facility, but women living below the poverty line also receive cash for deliveries outside of health facilities for their first two births.
4	Red de Proteccion Social	Nicaragua	Cash transfer were transferred to the mother in the beneficiary household for under the folowing health conditions of (1) bringing her children to scheduled preventive health care appointments—once a month for children under 2 years of age, and bimonthly (every other month) for those between two and five; (2) attending bimonthly health educational workshops and; (3) ensuring adequate weight gain for her children.
5	Manicaland HIV/STD Prevention Project	Zimbabwe	Children younger than 5 years linving in CCT houselholds had to be up-to-date with vaccinations and attend growth-monitoring clinics twice a year.

In regard to the health outcomes presented, the majority of the studies have presented biochemical or biometric health outcomes, including height-for-age, weight-for-age, weight-for-height and BMI-for-age in z-scores, birth weight, prevalence of stunting, wasting, overweight and hemoglobin concentration (*n* = 9). For the case of morbidity, one study used the incidence of diarrhea and acute respiratory infections, while six papers focused on child mortality indicators (perinatal, infant, neonatal and one-day mortality). In the case of health care services utilization, child immunization and vaccination coverage were mainly adopted in the studies selected for our final sample.

Although the majority of the studies reported poverty as the main children’s vulnerability for participating in a CCT program (*n* = 16), it was possible to identify other SDHs in the households of the beneficiaries, impacting the conditions in which their children are born, grow, work, live and age [10].

Households that were CCT beneficiaries were associated with lower educational level for one or both parents or the head of household (*n* = 5). CCT participants were also likely to live in an underprivileged location (*n* = 5), such as rural areas and minority groups regions. Gender issues (*n* = 1) were also associated with participants, including the existence of more female-headed households. Other SDHs of the participants were related to age (elderly-headed households, older women and child-headed households), race and ethnicity, coping with people with illness and disabilities and having or being an orphan in the household (Table 1).

The effects of CCTs on children’s health outcomes under the cited SDH were mostly positive. Studies that used immunization rates or vaccination coverage reported that CCT programs “led to a significant increase in childhood immunization rates” especially in high-priority locations (most vulnerable) in India [20] and the vaccination coverage increased more than 95% in Nicaragua for “children who live further away from a health facility or whose mothers are less educated” [21]. In Africa, the CCT arm of the randomized trial of a cash transfer program in Zimbabwe also showed an increase in up-to-date vaccination for children under 5 years old [22]. Improvements in child morbidity were also reported in Mexico where the incidence of diarrhea and acute respiratory infections has been reduced because of the participation in the CCT [23]. In the case of diarrhoeal diseases, the program had a significant positive effect among children in the most deprived households [23].

Nevertheless, the effects of CCTs were mixed for the child mortality indicators and biochemical or biometric health outcomes. Many of the CCTs in the present study reported reduction of infant, perinatal and postnatal mortality [24–27]. In Mexico, for example, larger reductions in neonatal and infant mortality were among groups with higher illiteracy rates and reduced access to electricity [24]. Studies concerning CCTs in India had conflicting results for the same indicators [20, 28]. The main explanation for this may be the difference in study designs and methods of these papers [20].

Biochemical or biometric health outcomes had also shown opposite results. In the case of Bolsa Familia, children enrolled in the program were more likely to have normal height-for-age and weight-for-age z-scores than non-beneficiaries, being both groups from impoverished areas [29]. Nonetheless, two other assessments of nutritional outcomes for children under 5 years old showed that no significant differences were found for underweight and stunting between children participants and non- participants of this CCT program [30]. These indicators were also not different from the child population of Brazil [31]. In both studies, authors prompted for more actions in health education in order to enhance population awareness of better food and nutrition practices.

## Discussion

The present integrative review was conducted in order to investigate if CCT programs create equality of opportunity in health for children under 5 years old. This particular life stage was chosen because it is critical for human development [13], which is the main purpose of traditional CCT programs. In order to identify the creation of equality of opportunity in health the present study applied Roemer’s conceptual framework of equality of opportunity and its capacity to mitigate the effects of unfair inequalities on health outcomes [6].

Although the selected studies applied a myriad of approaches and broad definitions for SDH, the literature review identified some trends in the creation of equality of opportunity in health for children by CCTs. First, we identified that CCTs created health opportunities for children because there were improvements in the health status of children with a vulnerable SDH. Nevertheless, creation of equality of opportunity in health for children was more reliable in quasi-experimental and experimental studies, considering that these study designs are more able to reduce causality bias.

In addition to this, it should be noticed that differences in the implementation phase, features and contexts of CCT programs could have affected the study outcomes. For example, Bolsa Familia, the Brazilian CCT, does not have health education lectures’ attendance as a condition for the participants receiving the cash transfer, even though these activities are seen as an important tool for the adoption of healthier behavioral practices [32]. On the other hand, Brazil has a large school feeding program in public schools that could complement the potential CCT effects on health and nutrition.

Income transfers alone or the use of conditional mechanisms to improve health may not be able to mitigate health inequalities in the presence of poor access to health services. Therefore investment in the supply-side of health services in the geographic locations targeted by the CCTs would improve health outcomes [33].

In addition, it should also be noticed that CCTs were mainly created for poverty reduction and development. Since many of the vulnerable groups are negatively affected by health inequities, the lack of access to financial resources could limit improvements in health status [33].

From a SHD perspective, income transfer alone is insufficient to mitigate unfair health inequalities because there is also a need to empower the most vulnerable and marginalized groups [34]. Although there is evidence of women empowerment through the participation of CCTs [35] and important advances in social inclusion [5], CCTs could inhibit social participation since the large-scale programs operate with a top-bottom approach in which governments dictate the eligible criteria and cash transfer conditions without any participation of the targeted population. Therefore, hierarchy powers in the case of CCTs could undermine the control that individuals and communities have over their lives [36].

### Study limitations

Three main limitations of the present literature review need to be acknowledged. First, although the methods for searching the literature were systematic, it is not prudent to guarantee that all relevant studies on the CCTs and children’s health outcomes have been identified. There was a limited number of languages and electronic bibliographical databases adopted in the study. In addition to this, our literature review used only second-handed data, so publication bias could be an issue. Third, we have not included gray literature, which have a considerable body of literature regarding CCTs and child health and represent an important source of information.

## Conclusions

CCTs seek to create life chances for children to overcome poverty and exclusion, thus reducing inequality of opportunity. We identified that CCTs created health opportunities for children, even though there was a variety of study design and methods that made it a challenge to compare study results.

The creation of health opportunities for children by the CCTs could have positively impacted health inequalities, but these results are reduced in face of poor health services and limited social participation of beneficiaries in the decisions regarding the implementation and conditions of the program.

Moreover, we noticed that there is a lack of methodological research focused in describing the mechanisms of how this equality of opportunity functions, especially in health. Our study tried to fulfill this gap by suggesting a definition for the creation of equality of opportunity in health based on the case of CCTs. In depth theoretical studies regarding this concepts are important to improve the construction of a framework to guide policy decisions in health.

## Additional file

Additional file 1: Detailed search strategies. (DOCX 19 kb)
