# Supplementary material for: Conditional cash transfers and the creation of equal opportunities of health for children in low and middle-income countries: a literature review
Source: Int J Equity Health. 2017 Aug 31;16:161. doi: 10.1186/s12939-017-0647-2 (PMC5580215; doi:10.1186/s12939-017-0647-2)
Supplement: Additional file 1: — Detailed search strategies. (DOCX 19 kb) [file 12939_2017_647_MOESM1_ESM.docx]

**Additional file 1.** Detailed search strategies.

1. **Academic Search Complete (EBSCO)**

Search date February 20 2017

1. Advanced search: ("cash transfer" OR "conditional cash transfer" OR CCT OR “income transfer”) AND (child OR children OR health OR “child health” OR "health outcomes" OR “health promotion” OR nutrition ) AND (opportunit* OR "equality of opportunity" OR equality OR equity OR poverty OR inequality) 124

**Limiters** – Full text Publication Year: January of 2006- June of 2016 124

**Search modes** - Boolean/Phrase 124

**Search results:**

| **#** | **Searches** | **Results** |
| --- | --- | --- |
| 1 | ("cash transfer" OR "conditional cash transfer" OR CCT OR “income transfer”) AND (child OR children OR health OR “child health” OR "health outcomes" OR “health promotion” OR nutrition) AND (opportunit* OR "equality of opportunity" OR equality OR equity OR poverty OR inequality) | 124 |

1. **Pubmed**

Search date February 9 2017

1. ("cash transfer" OR "conditional cash transfer" OR CCT OR “income transfer”) AND (child OR children OR health OR “child health” OR "health outcomes" OR “health promotion” OR nutrition ) AND (opportunit* OR "equality of opportunity" OR equality OR equity OR poverty OR inequality) 207

| **#** | **Searches** | **Results** |
| --- | --- | --- |
| 1 | ("cash transfer" OR "conditional cash transfer" OR CCT OR “income transfer”) AND (child OR children OR health OR “child health” OR "health outcomes" OR “health promotion” OR nutrition) AND (opportunit* OR "equality of opportunity" OR equality OR equity OR inequality) | 207 |

1. **Scopus**

Search date February 6 2017

1. TITLE-ABS-KEY ("cash transfer" OR "conditional cash transfer" OR CCT OR “income transfer”) AND (child OR children OR health OR “child health” OR "health outcomes" OR “health promotion” OR nutrition) AND (opportunit* OR "equality of opportunity" OR equality OR equity OR poverty OR inequality) 1152

**Limiters** – Year: 2006 – 2016 1022 Document Type: Article, Review 924 Language: English, Portuguese and Spanish 913

**Search modes** – Advanced search

| **#** | **Searches** | **Results** |
| --- | --- | --- |
| 1 | ("cash transfer" OR "conditional cash transfer" OR CCT OR “income transfer”) AND (child OR children OR health OR “child health” OR "health outcomes" OR “health promotion” OR nutrition) AND (opportunit* OR "equality of opportunity" OR equality OR equity OR poverty OR inequality) | 913 |

1. **Web of Science**

Search date February 20 2017

1. Topic: ("cash transfer" OR "conditional cash transfer" OR CCT OR “income transfer”) AND (child OR children OR health OR “child health” OR "health outcomes" OR “health promotion” OR nutrition ) AND (opportunit* OR "equality of opportunity" OR equality OR equity OR poverty OR inequality) 205

**Timespan – Year:** 2006-2016 205

**Refine results**- Type of papers: Articles, Review 200 Language: English, Portuguese and Spanish 199
